# Evidence of impaired H-reflex and H-reflex rate-dependent depression in diabetes, prediabetes and obesity: a mini-review

**DOI:** 10.3389/fendo.2023.1206552

**Published:** 2023-07-05

**Authors:** Rebeca Kababie-Ameo, Gabriela Gutiérrez-Salmeán, Carlos A. Cuellar

**Affiliations:** ^1^ Facultad de Ciencias de la Salud, Universidad Anáhuac Mexico, Huixquilucan, Estado de Mexico, Mexico; ^2^ Centro de Especialidades del Riñon (CER), Naucalpan de Juarez, Estado de Mexico, Mexico; ^3^ Escuela de Ciencias del Deporte, Universidad Anahuac Mexico, Huixquilucan, Estado de Mexico, Mexico

**Keywords:** H-reflex, rate-dependent depression, polyneuropathy, diabetes, prediabetes, obesity

## Abstract

Diabetes Mellitus is a public health problem associated with complications such as neuropathy; however, it has been proposed that these may begin to develop during prediabetes and may also be present in persons with obesity. Diabetic peripheral neuropathy is the presence of signs and/or symptoms of peripheral nerve dysfunction in people living with diabetes, which increases the risk of developing complications and has a deleterious impact on quality of life. As part of the therapeutic protocol for diabetes, screening tests to identify peripheral neuropathy are suggested, however, there are no recommendations for people with prediabetes and obesity without symptoms such as pain, numbness, or paresthesias. Moreover, clinical screening tests that are usually used to recognize this alteration, such as tendon reflex, temperature sensation, and pressure and vibration perception, might be subjective as they depend on the evaluator’s experience thus the incorrect application of these tests may not recognize the damage to small or large-nerve fibers. Recent evidence suggests that an objective study such as the impairment of the rate-dependent depression of the H-reflex could be used as a biomarker of spinal disinhibition and hence may provide more information on sensorimotor integration.

## 1 Introduction

Diabetes Mellitus (DM) is a public health problem which, according to the International Diabetes Federation in 2021, affects 537 million people; in addition, it is estimated that 541 million people have impaired glucose tolerance thus incidences are expected to increase in the coming years. This disease represents a high economic burden and its direct health expenditures are expected to be close to one trillion USD (1). DM is associated with macro and microvascular complications; the former involves cardiovascular diseases, i.e., of the large blood vessels, while the latter includes retinopathy, nephropathy, and diabetic neuropathy (2). Diabetic neuropathy is a highly prevalent complication of DM, with a complex pathophysiology, associated with pain and symptoms such as dysesthesias, numbness, hyperalgesia, allodynia, and ataxia (3, 4).

Prediabetes is defined as the condition when subjects do not yet meet the criteria for diabetes but have an alteration in the carbohydrate metabolism, which can be identified by glycated hemoglobin (HbA1c) between 5.7 and 6.4% and/or fasting plasma glucose from 100 to 125 mg/dL and/or impaired glucose tolerance, evidenced by plasma glucose between 140 and 199 mg/dL after 2 hours of a 75 g oral glucose tolerance test. Its relevance lies in the fact that it increases the risk of cardiovascular diseases and the progression of type 2 DM (T2DM), actually, macro and microvascular complications of diabetes may begin to develop during prediabetes (5).

A study conducted in Germany included 844 patients with T2DM, reported that 35% had Diabetic sensorimotor polyneuropathy (DSPN) within the first year of DM diagnosis (6). In a systematic review, it was stated that up to 77% of people living with prediabetes may have peripheral neuropathy. This led to the conclusion that in some patients, neuropathy affects initially small nerve fibers and continuous or episodic pain can be present and microvascular complications may be detected even before the progression to T2DM. However, even though it is recognized that neuropathy may be present in a prediabetes state, screening is usually only recommended when patients show symptoms (6–8).

In addition to hyperglycemia, other cardiometabolic risk factors have been proposed to contribute to microvascular dysfunction, including central obesity, which has been associated with worse nerve function and is considered the second most important metabolic risk factor for neuropathy (9, 10). Callaghan et al., reported in 2 studies that neuropathy was present in 2.2 to 3.8% of lean controls, 11.1 to 12.1% in subjects with obesity but normoglycemia, 7.1-29% in participants with obesity and prediabetes and 34.6 to 40.8% in subjects with obesity and diabetes. In one of the studies, a multivariable logistic regression associated waist circumference with neuropathy (OR=1.39 95%CI 1.10-1.75) (11, 12).

Excessive adiposity has been correlated with loss in small unmyelinated axons and more predisposition to injury, suggesting that obesity might injure, especially, small-nerve fibers, secondary to microvascular and peripheral nerve injury. It has also been proposed that the association with neuropathy may be mediated by inflammation (9, 13).

It has been reported that both small and large-diameter nerve fibers are affected but small nerve fibers are considered to be the first involved in DSPN (14). Within small-diameter fibers, unmyelinated C fibers and thinly myelinated A delta (Aδ) fibers are responsible for temperature and pain perception, thus, to assess the function of these nerve fibers, testing temperature and sharp sensation or pinprick is recommended (15, 16). In contrast, large fibers functions are related to vibration sensation, pressure perception, and proprioception, and its evaluation through the tuning fork and monofilament is required. These are the most common screening tests used in clinical practice but a broader screening is crucial (3, 15). Diabetic neuropathies are a heterogeneous group of disorders affecting peripheral and autonomic nerves and are associated with reduced health-related quality of life and a high economic burden. They are the most common complication of DM, and worldwide, it is estimated that 50% of people living with DM develop this complication. Among diabetic neuropathies, DSPN is the most prevalent and is defined as the presence of signs and/or symptoms of peripheral nerve dysfunction in people living with DM after excluding other causes (8, 17). DSPN is a common complication in people living with diabetes and it is defined as the presence of signs and/or symptoms of dysfunction of the peripheral nerves due to chronic hyperglycemia, associated metabolic derangements, and other cardiovascular risk factors such as dyslipidemias and obesity (18, 19).

DSPN includes symptoms amidst pain, dysesthesias, numbness, hyperalgesia, and allodynia. However, up to 50% of people with DSPN may be asymptomatic, thus increasing the risk of injuries and lower limb amputations (15). The diagnosis of DSPN is made by exclusion, so it is necessary to evaluate the other causes such as vitamin B12 deficiency, hypothyroidism, inherited neuropathies, vasculitis, or neuropathy as a consequence of alcohol or chemotherapy (3). Once these causes are excluded, medical and general history, feet examination and neurological inspection are fundamental to identify the severity of the DSPN and the possible affected fibers and with it, the risk of injuries and specific complications (15). As a part of the neurological inspection, it is important to assess small and large fibers with clinical tests such as vibration and pressure perception (using a 128-Hz tuning fork and a 10g Semmes-Weinstein monofilament, respectively), temperature and pinprick sensation, and knee and ankle reflexes (20). However, these tests rely on the subject’s perception, and subjective scores may affect diagnosis.

Electrophysiological tests such as the Hoffmann reflex (H-reflex) provide useful information about the spinal somatosensory function. The H-reflex is the electrical-induced analog to the mechanical myotatic reflex of the muscle spindle that generates afferent and efferent components. It is produced by percutaneous electrical stimulation of a peripheral nerve and recorded in a determined muscle. The H-reflex evaluates the excitability of the alpha-motoneurons (αMN) when presynaptic inhibition and intrinsic excitability of the αMN remain constant (21, 22). Within the properties of the H-reflex, a prolonged latency may be an indicator of neuronal damage (23). Other relevant component of the H-reflex is the ratio of the amplitude of the maximum H-reflex to the maximum M-wave, which can provide information about the excitability of αMN (23, 24).

On the other hand, the Rate-Dependent Depression of the H-reflex (RDD hereinafter) is observed when the amplitude of the H-wave over paired or consecutive stimulations at frequencies > 1 Hz decreases compared to the first evoked response (25). Recently, the use of the RDD has been proposed to evaluate somatosensory processing in the spinal cord since its impairment is associated with diabetic neuropathy in animal models (26–28) and patients with type 1 and 2 diabetes (26, 29–31).

This mini-review describes the main findings of alterations of the H-reflex and the RDD, the latter being proposed as evidence of spinal disinhibition in painful diabetic neuropathy, and recently as a complementary screening tool to assess DSPN in subjects with glycemic impairment (diabetes and prediabetes) and in obesity. Importantly, results are restrained to electrophysiological reports made in lower limbs. Finally, as the impairment of the RDD has gained attention, cellular and molecular features of this phenomenon are also provided.

## 2 Alterations of the H-reflex and H-reflex rate-dependent depression in diabetes, prediabetes, and obesity

Alterations in electrophysiological parameters assessed during H-reflex testing have been reported in early-diagnosed diabetic patients classified as normoglycemic (NG) and hyperglycemic (HG), for example, the amplitude of the H-reflex decreased significantly in NG and HG subjects compared to healthy controls, although latency did not show significant changes. Prolonged latencies of the M-wave were observed in 58%, while amplitude diminished in 12% of the sample; however, no significant differences in amplitude nor latency were found between groups (32). In the search for an early electrophysiological marker for subclinical neuropathy, Marya et al. (33), found a significantly increased H-reflex latency in diabetics compared to controls (p < 0.001). Interestingly, authors found alterations in 85% (11/13) of early-onset diabetes subjects (onset between 20-40 years). Zhou et al. (34), also found a significant increase in H-reflex latency in the non-painful diabetic neuropathy group (p-DPN) and painful diabetic neuropathy group (p+DPN) compared to controls (p < 0.017). Salinas et al. (29), reported that the T2DM group presented a significantly increased latency compared to the control group (p < 0.001), and when subgroups were analyzed for p-DPN and p+DPN, latencies were significantly prolonged in the non-painful group at all stimulation frequencies. Also, Marshall et al. (26), found that latencies were prolonged in p-DPN and p+DPN groups vs control subjects (p < 0.05 and p < 0.01, respectively).

In addition to the H-reflex test, electrophysiological studies such as nerve conduction velocity (NCV), help to diagnose large fiber neuropathy but are less specific for small fibers, so, small fiber neuropathy could be underdiagnosed (20), thus, other tests have been proposed such as the RDD which has been suggested as a biomarker of spinal disinhibition associated with pain in DSPN and which does not assess large or small fibers, but rather somatosensory dysfunction (29). Interestingly, it has been observed that the RDD is not only impaired in DSPN but also in overweight and obesity with metabolic syndrome (29). Therefore, this test may be helpful as a complementary tool when searching for DSPN even in the absence of pain or a diabetes diagnosis (13).

Marshall et al., studied DSPN in T1D and compared the RDD in 3 groups: p+DPN, p-DPN, and a control group, and found that the amplitude of the H-wave was reduced in both groups, but just the p+DPN group presented a significant impairment (loss) of the RDD. Moreover, patients with higher somatosensory thresholds had greater RDD impairment (although not significant), suggesting that spinal inhibitory dysfunction may be the mechanism contributing to pain. In addition, corneal nerve fiber density was significantly lower in both p-DPN and p+DPN groups compared to controls, but even lower in the p+DPN group (26). In addition, Marshall et al., reported that levels of RDD impairment were associated with classical symptoms of DSPN such as mechanical pain sensitivity, heat hyperalgesia, and spontaneous burning pain in T1D and T2DM, representing a “hyperpathia phenotype” related to spinal disinhibition (35). In two studies from the same research group, Worthington et al. (30, 31), reported that in patients living with T1D and T2DM, RDD was impaired in p+DPN, but surprisingly, patients with p-DPN presented enhanced RDD, even compared to controls, not observed in a previous report [see Figure 4 in Marshall et al., 2017 (26)]. The former authors suggested that RDD in p-DPN could be increased to suppress peripheral nociceptive inputs that may contrarily cause pain (31). A study performed by Zhou et al. (34), found a greater impairment of RDD in the p+DPN group compared to controls and p-DPN. For instance, at 1 Hz, the p+DPN group had a RDD of 17.9% compared to 41.9% and 31.7% of the p-DPN and control groups, respectively. In the same study, it is reported an improvement of 7.5% in the RDD after 2 weeks of gabapentin treatment (34). Nevertheless, Salinas et al., reported that although there is greater impairment of RDD in subjects with painful neuropathy, RDD is also impaired in subjects with non-painful diabetic neuropathy and they did not find enhanced RDD in any subpopulation of T2DM subjects (29). A common observation between both research groups is that H-reflex might be absent or severely attenuated in some patients, specifically in those with severe DSPN, so this test may not be suitable for all subjects, being limited to mild and moderate neuropathy (26, 29, 31).

The RDD has not been extensively studied in subjects with prediabetes yet; however, Salinas et al., reported that subjects with HbA1c levels considered as prediabetes exhibited significantly longer H-reflex latencies compared to controls and impaired RDD at different stimulation frequencies. These findings suggest that evaluation of the RDD may be used to identify DSPN in conditions such as prediabetes (29). On the other hand, few studies have evaluated the RDD in conditions of overweight and obesity but their results are interesting. In a preclinical study (36), male and female mice were fed with a high-fat diet (HFD) and researchers measured the RDD at 5 Hz at weeks 5, 10, and 15, and observed that the HFD male group had impaired RDD at weeks 5 and 10, while the female group had impaired RDD at weeks 10 and 15, compared to the control group. However, in this study, behavioral measures of pain were not assessed to determine if worse scores were related to impaired RDD (36). In humans, Salinas et. al., subdivided the control group according to their BMI and found that almost 50% of the subjects with overweight and obesity and without a diagnosis of DM, had impaired RDD at 1, 2, 5, and 10 Hz, unfortunately, there was no data available in this study including biochemical markers suggesting metabolic disorders (29).

Finally, when accompanied by clinical tests such as the Michigan Neuropathy Screening Instrument, absence or prolonged latency of the H-reflex were significantly associated with a predictive value for DSPN (OR 4.3; 95% CI 1.6, 11.2) (37). However, the measurement of the H-reflex alone has some limitations, for example, its magnitude can change during muscle contraction or incorrect posture; i.e., the M-wave amplitude should remain stable to stimulate a constant number of motor nerve fibers and to maintain the excitability of Ia afferents and the H-wave diminishes as the stimulus intensity increases (22, 38). Besides, it’s been reported by different authors that H-reflex is absent in some people living with diabetes (29, 31, 32).

## 3 Cellular and molecular features of the RDD

In rodent models of diabetes and metabolic syndrome, spinal disinhibition is the pro-nociceptive alteration involving a reduction in potassium/chloride co-transporter (KCC2) in the dorsal spinal cord and the consequent elevation of intraneuronal chloride concentration (30, 39). The shift of the transmembrane anion gradient causes the binding of γ-aminobutyric acid (GABA) to post-synaptic GABA_A_ receptors and generates an outflow of chloride ions and membrane depolarization, and the neurotransmitter GABA switches from an inhibitory role to an excitatory function (40).

In experimental diabetes, reduced spinal KCC2 expression in rats was associated with impaired RDD as well as allodynia; these both alterations were GABA_A_ dependent (40). In another study with naïve rats, spinal inhibition of KCC2 also resulted in allodynia, thus, is suggested that this symptomatology is a consequence of the excitatory function of GABA (39, 40). Another study in rats showed that streptozotocin-induced tactile allodynia and intrathecal injection of L-655,708 (a selective inverse agonist for the benzodiazepine site of GABA_A_ receptors containing the α5 subunit), provoked an antiallodynic effect, suggesting that extrasynaptic α_5_GABA_A_ receptors found in the spinal cord, play a pronociceptive role in diabetic rats, and also, this injection of L-655,708 restored RDD in the group of diabetic rats (28).

It is proposed that, when there is a lack of depression of the RDD, in p+DPN, spinal inhibition may be altered, with an excitatory GABAergic function instead of inhibition, so a potential application could be the use of this study to identify patients who may benefit from therapies directed at spinal disinhibition (31, 41).

Although RDD has been studied for diabetic neuropathy, it has been reported that neuropathy may be present in people with prediabetes and obesity, therefore it may be helpful as an auxiliary test to identify the development of this complication even before the onset of diabetes mellitus.

## 4 Discussion

DSPN is a high-prevalence complication associated with reduced health-related quality of life, which, in turn, is related to further complications such as foot ulceration and lower-limb amputations, therefore, early diagnosis is essential (42). Additional to DM, there are reports of peripheral neuropathy in prediabetes (13), suggesting that long-term microvascular complications of DM may begin to develop since the prediabetic state. Importantly, DSPN is present in 35% of patients at the time of diagnosis of T2DM (7). Also, excessive adiposity has been recognized as a risk factor for neuropathy independently of glucose level alteration but when combined with prediabetes or diabetes, the risk increases even more (11, 12). Different clinical screening tests and studies might help to identify small and large-nerve damage and among them are vibration and pressure perception, temperature sensation, pinprick and knee and ankle reflexes, but also nerve conduction studies, and recently, the RDD (20, 31). This test may be used to evaluate somatosensory processing (i.e. spinal disinhibition) not only in diabetes but also in painless neuropathy and prediabetes. The RDD could be also important for identifying specific therapies that might help a target group of patients (29, 31).

The RDD has been studied in rodent models of diabetes and subjects with T1D and T2DM, but the stimulation parameters such as the number of pulses and stimulation frequency, to obtain the best and most precise results are still under investigation, as well as the cut-off point to determine the presence, or not, of an alteration related to painful neuropathy (30). Research groups studying RDD have reported similarities related to impaired RDD in painful diabetic neuropathy, on the other hand, differences have arisen, specifically, related to the impairment of RDD in patients with painless diabetic neuropathy (26, 29, 30, 35).

Finally, it is worth noting that differences between males and females in terms of pain assessment and % of RDD have not been addressed yet (43).

Due to the presence of comorbidities and pharmacological treatment in cohorts, interpretation of impaired RDD must be taken cautiously. In conclusion, RDD of the H-reflex is a non-invasive, objective study that may be useful to identify the presence of spinal disinhibition related to neuropathy in diabetes, prediabetes, and obesity. RDD reflects somatosensorial processing and current evidence suggests that this is independent of the degree of small and large fibers damage. However, H-reflex may not be present in all patients, specifically in severe diabetic neuropathy, and to date, there is no consensus about the methods and cut-off points that should be considered when performing this electrophysiological test.

## Author contributions

RKA, GGS, and CAC conceived of the presented idea and RKA wrote the manuscript with support from GGS and CAC, and CAC revised the work critically for important intellectual content. All authors contributed to the article and approved the submitted version.
